# Mass spectrometry-based proteomics profiling of dogs with and without oral diseases: a systematic review

**DOI:** 10.1186/s12903-024-04096-x

**Published:** 2024-03-22

**Authors:** Paras Ahmad, Walter L. Siqueira

**Affiliations:** College of Dentistry, University of Saskatchewan, 105 Wiggins Road, Saskatoon, SK S7N 5E5 Canada

**Keywords:** Biomarkers, Canine, Dog, Mass spectrometry, Proteomics

## Abstract

**Background:**

Understanding the distinct proteomics profiles in dogs’ oral biofluids enhances diagnostic and therapeutic insights for canine oral diseases, fostering cross-species translational research in dentistry and medicine. This study aimed to conduct a systematic review to investigate the similarities and differences between the oral biofluids’ proteomics profile of dogs with and without oral diseases.

**Methods:**

PubMed, Web of Science, and Scopus were searched with no restrictions on publication language or year to address the following focused question: “*What is the proteome signature of healthy versus diseased (oral) dogs’ biofluids?*” Gene Ontology enrichment and the Kyoto Encyclopedia of Genes and Genomes pathway analyses of the most abundant proteins were performed. Moreover, protein-protein interaction analysis was conducted. The risk of bias (RoB) among the included studies was assessed using the Joanna Briggs Institute (JBI) Critical Appraisal Checklist for Studies Reporting Prevalence Data.

**Results:**

In healthy dogs, the proteomic analysis identified 5,451 proteins, with 137 being the most abundant, predominantly associated with ‘innate immune response’. Dogs with oral diseases displayed 6,470 proteins, with distinct associations: ‘defense response to bacterium’ (periodontal diseases), ‘negative regulation of transcription’ (dental calculus), and ‘positive regulation of transcription’ (oral tumors). Clustering revealed significant protein clusters in each case, emphasizing the diverse molecular profiles in health and oral diseases. Only six studies were provided to the JBI tool, as they encompassed case-control evaluations that compared healthy dogs to dogs with oral disease(s). All included studies were found to have low RoB (high quality).

**Conclusion:**

Significant differences in the proteomics profiles of oral biofluids between dogs with and without oral diseases were found. The synergy of animal proteomics and bioinformatics offers a promising avenue for cross-species research, despite persistent challenges in result validation.

**Supplementary Information:**

The online version contains supplementary material available at 10.1186/s12903-024-04096-x.

## Background

Within the Canidae family, domestic dogs constitute a species that is extensively distributed [1]. This species assumes a crucial role as a companion to humans and serves as an interventional support for various mental illnesses and physical disabilities, including sight or hearing impairment, and autism, among other conditions [2, 3]. Given their shared environment with humans, dogs have emerged as valuable models for researching various diseases, including cognitive aging [4], gene therapy for genetic diseases [5], cardiovascular diseases [6], and cancer [7].

The terminology *proteomics* encompasses the comprehensive study of proteins, encompassing their functions and structures. In contrast, the *proteome* is the complete set of proteins expressed by the genetic material of an organism under specified environmental conditions [8]. Proteomics has rapidly emerged as a research field in less than 20 years [9], experiencing swift development propelled by advancements in technology and the imperative for analytical approaches capable of providing comprehensive characterization of proteins on a global scale. The capacity to sequence complete genomes and organize the ensuing data into genome sequences has facilitated proteomics. However, achieving global characterization of the proteins constituting even relatively simple biological systems remains elusive [10]. Typically, a proteome is more intricate than the encoding genome, with proteins spanning a broad dynamic range in terms of their presence and abundance [11]. These challenges are compounded by the regulation of protein expression in response to developmental and environmental stimuli, leading to a dynamic proteome. However, the significance of proteins as the principal effector molecules in biology, serving as primary antigens and drug targets, has generated considerable interest and investment in proteomics. Consequently, the field continues to undergo rapid development [12].

In recent years, there has been a growing interest in utilizing proteomics and complementary advancements in bioinformatics to tackle issues related to veterinary pathogenesis. However, the application of proteomics in veterinary dentistry has been somewhat constrained compared to studies exploring the potential of advanced protein-analytic technologies in human clinical dentistry [12].

Over the past two decades, there has been significant evolution in the application of mass spectrometry (MS)-based approaches to identify and characterize proteins in veterinary dentistry [13]. The prerequisite for applying MS in animal proteomics has been the whole genome sequencing of important animal species in veterinary sciences, including but not limited to dogs, cats, horses, sheep, cows, pigs, and chickens [14]. Despite significant advancements in commercially available MS instrumentation, marked by improvements in wide dynamic range, molecular specificity, resolution, and high sensitivity, the primary challenges in the field of veterinary proteomics persist. These challenges revolve around incompletely characterized animal genome sequences, as well as incomplete Gene Ontology (GO) annotations and mapped pathways, posing obstacles to the study of non-model organisms [15, 16]. One approach to address this challenge is employing a homology-driven method in both bioinformatic analyses and database searches, or conducting *de-novo* sequencing to identify protein [13].

Various studies have explored proteomic approaches to understand oral health and disease in dogs. Pisamai et al. [17] characterized protein expression profiles in oral tumors, unveiling potential biomarkers and their associations with chemotherapy drugs. Davis et al. [18] investigated proteomic changes in gingival crevicular fluid (GCF) during periodontal disease progression. Ploypetch et al. [19] focused on salivary biomarkers for early detection of oral tumors, validated through western blot analysis. Later, the same group [20] examined salivary biomarkers for oral tumors, emphasizing PTPN5 and p53. Similarly, Ploypetch et al. [21] explored salivary biomarkers for monitoring therapeutic response in canine oral melanoma, discovering a potential prognostic biomarker. Recently in 2022, Ploypetch et al. [22] investigated the composition of acquired enamel pellicle on canine teeth, revealing proteins involved in bacterial colonization. In 2020, Bringel and colleagues [23] characterized saliva proteomics in dogs with and without dental calculus, identifying potential biomarkers for periodontitis. These studies collectively enhance understanding of canine oral health, offering insights into diagnosis, treatment monitoring, and disease mechanisms.

While the utilization of proteomics in veterinary dentistry has trailed behind its use in human dentistry, there has been a recent uptick in activity, particularly in the investigation of health and disease in farm animals [12]. Several pertinent and informative reviews have been published that establish the foundation for increased involvement of veterinary laboratories in this dynamic and rapidly advancing field [24–28]. While the number of studies on proteomics in dogs is comparatively fewer than those conducted in humans, research in this area has been reported [29–31]. In these canine studies, saliva and serum are the most frequently utilized sample types [32–34], with fewer investigations conducted on other sample types such as GCF. Given the diverse and extensive nature of literature regarding technical applications and the various pathologies studied in the canine species, systematic reviews on this topic can be highly valuable in consolidating and synthesizing this knowledge.

In the realm of canine research, three prior reviews have underscored the significance of proteomic analysis [35–37]. Furthermore, another review delved into the applications of proteomics specifically in dogs with cancer [38]. Hence, the present study aimed to conduct a systematic review for investigating the similarities and differences between the oral biofluids’ proteomics profile of dogs with and without oral diseases.

## Methods

### Study protocol and registration

This scoping review adhered to the PRISMA (Preferred Reporting Items for Systematic Reviews and Meta-Analyses) guidelines [39] and was officially registered in the Open Science Framework (OSF; 10.17605/OSF.IO/D2GWA).

### Eligibility criteria and focused question

The eligibility criteria were based on the PICO format: Population (P) included healthy dogs without any oral and systemic diseases; intervention (I) included biofluids including saliva, GCF, or blood serum; Comparison (C) included dogs with oral disease; and Outcome (O) included proteomics analysis involving compilation of expressed proteins using MS. For the present study, the following focused question was formulated based on the PICO format [40]: “*What is the proteome signature of healthy dogs versus diseased (oral) dogs’ biofluids?*”

This systematic review included experimental (clinical trial) or observational (case-control) studies without limitations on publication language or year. Exclusions comprised studies: (i) lacking the reporting of protein signatures; (ii) absence of the application of MS; (iii) presence of disease other than oral or dental disease; and (iv) literature reviews, systematic reviews, meta-analyses, editorials, and case studies.

### Search strategy

A literature search was performed electronically, utilizing the following databases: PubMed (MEDLINE), Clarivate Analytics’ Web of Science (All Databases), and Elsevier’s Scopus, with no restrictions on publication language or year. A combination of the following free terms and Medical Subject Headings (MeSH) words was used in the title, abstract, and keywords section: “dog” OR “canine” AND “proteome” OR “proteomics” AND “mass spectrometry”.

We performed an extra search in the gray literature, covering sources such as OpenGrey (https://www.opengrey.eu) and Google Scholar. Moreover, a backward search was conducted subsequent to the screening process, wherein we scrutinized the reference lists of all included articles at that stage to identify cross-references. We repeated the search to identify any recently published studies that may have surfaced just before submitting this article.

### Literature screening strategy

The screening process utilized the Covidence review management tool (Veritas Health Innovation Ltd., Australia), which automatically conducted a duplicate check upon data import. After an initial calibration that encompassed aspects such as search terms, search databases, eligibility criteria, and the review management tool, two examiners (P.A. and W.L.S.) independently carried out the screening process. This included the initial assessment of titles and abstracts, followed by a thorough review of full-text articles. Upon completion of both phases, Covidence facilitated a conflict resolution procedure, during which discrepancies or conflicting decisions were discussed and resolved through discussion.

### Data extraction

The following data was extracted from the included studies: (i) study references and location; (ii) health status of study participants; (iii) sample size; (iv) gender distribution; (v) age of dogs in months; (vi) sample type; (vii) amount of sample collected in µL; (viii) time of sample collection; (ix) duration of sample collection; (x) method used to quantify total proteins; (xi) MS-based approach used; (xii) peptide labeling approach used; (xiii) protein verification approach used; (xiv) additional approaches used; (xv) false discovery rate [FDR] threshold used; (xvi) number of proteins identified; and (xvii) number of significant or most abundant proteins.

### Bioinformatics analysis

The most abundant proteins found in healthy as well as diseased dogs underwent Gene Ontology (GO) and Kyoto Encyclopedia of Genes and Genomes (KEGG) pathway analyses. This analysis utilized the Database for Annotation, Visualization, and Integrated Discovery (DAVID version 6.8) [41]. GO biological processes (BP), cellular compartments (CC), molecular functions (MF) terms, and KEGG pathways at the DIRECT level were filtered using the modified Fisher’s exact test, with a significance threshold set at a *p*-value less than 0.05. Visualization of the results, including GO enrichment and KEGG pathway analyses, was achieved through the website (https://www.bioinformatics.com.cn) to generate a bubble dot figure. By utilizing the Search Tool for the Retrieval of Interacting Genes/Proteins (STRING) database, a protein-protein interaction (PPI) network was formed and analyzed [42]. The cutoff criterion for required confidence (combined score) was set at > 0.4. Additionally, the Markov Cluster algorithm (MCL) [43] clustering was applied with a minimum of three inflation parameters to pinpoint the top three clusters.

### Risk of bias assessment

The utilization of the Joanna Briggs Institute (JBI) Critical Appraisal Checklist for Studies Reporting Prevalence Data [44] was solely employed for studies that encompassed dogs with oral disease(s). The risk of bias (RoB) assessment was conducted by two independent reviewers (P.A. and W.L.S.). The tool consisted of the following items: (i) was the sample representative of the target population; (ii) were study participants recruited in an appropriate manner; (iii) was the sample size adequate; (iv) were the research participants and the setting described in detail; (v) was the data analysis performed with adequate coverage of the identified sample; (vi) were objective and standard criteria utilized to measure the condition; (vii) was the disease measured reliably; (viii) was there appropriate statistical/data analysis; and (ix) are all important confounding factors, subgroups, differences identified and accounted for. The reviewers engaged in a discussion regarding the scoring, and a consensus was reached concerning the characterization of the applied methodology based on specific categories. The study was deemed as having a “high” score when it attained up to 49% with a positive response. For scores ranging from 50 to 69% with affirmative answers, the study was classified as “moderate.” Lastly, a score of more than 70% with positive responses placed the study in the “low” category [44].

## Results

### Search strategy outcomes

**Fig. 1:**
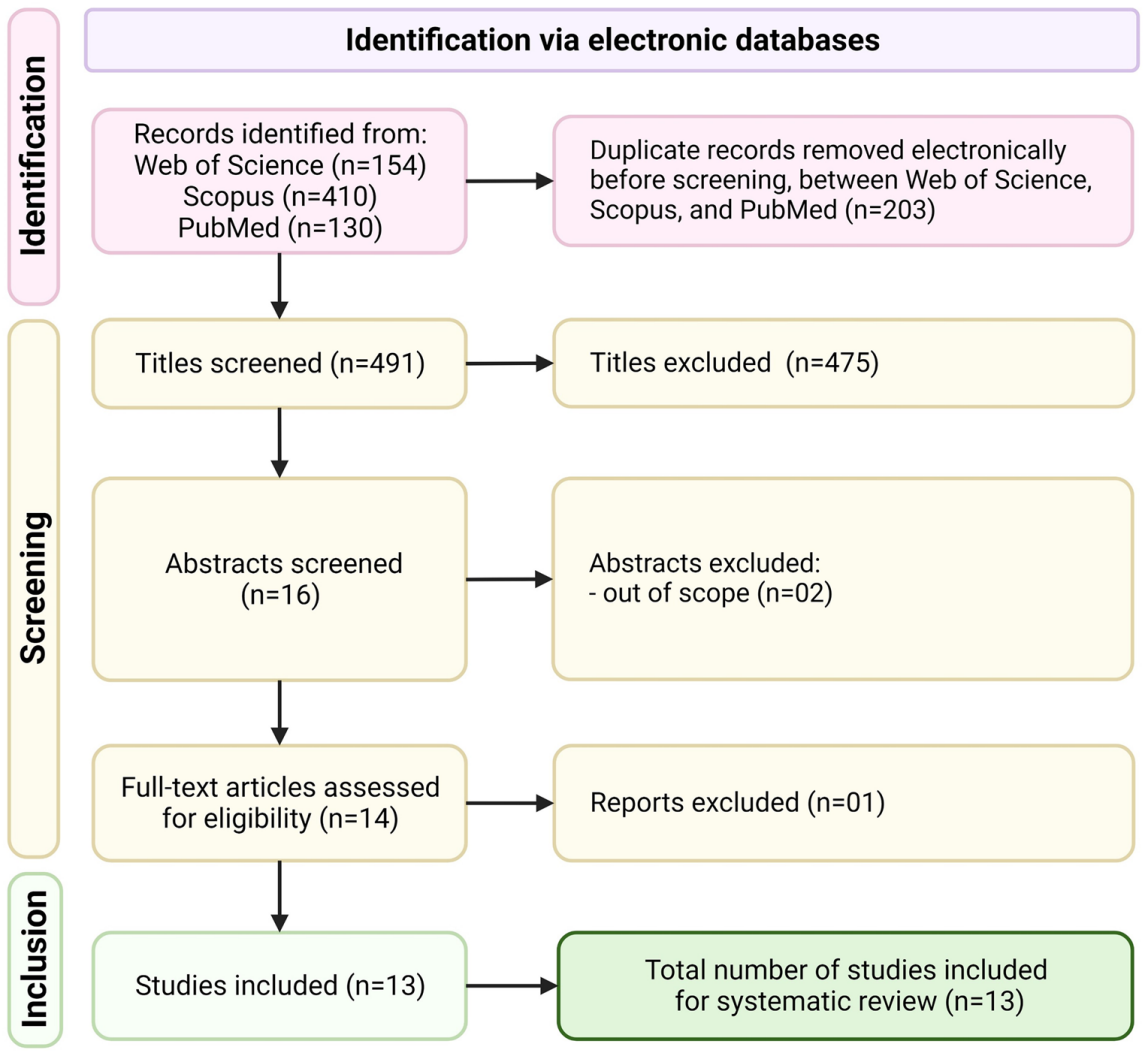
Literature search outcomes following the PRISMA guidelines [39]

The initial search strategy resulted in the identification of 694 articles via PubMed (*n* = 130), Scopus (*n* = 410), and Web of Science (*n* = 154) databases. After eliminating duplicate articles, a total of 491 articles were retained for analysis. Following the title screening, 475 articles were excluded. Then, 2 articles were removed after the abstract screening protocol [45, 46]. At this stage, full texts of the remaining 14 articles were assessed which resulted in the removal of one article [47]. Eventually, 13 articles were included in the present systematic review (Fig. 1) [17–23, 48–53].

### General features of included studies

MS-based proteomics studies of dogs with and without oral diseases were published between 2015 [52] and 2022 [22, 48], of which most of the investigations were performed in Thailand (*n* = 6) [17, 19–22, 49], followed by the United Kingdom (*n* = 3) [18, 48, 51], Portugal (*n* = 2) [52, 53], Canada (*n* = 1) [23], and the United States (*n* = 1) [18]. Overall, 439 dogs (range: 1–53; mean: 13) were utilized in the included studies, of which 172 were males and 154 were females. In total, 33 different breeds of dogs were used including Schnauzer (*n* = 54), mixed (*n* = 36), Beagle (*n* = 34), Labrador Retriever (*n* = 27), Poodle (*n* = 25), Golden Retriever (*n* = 21), Shi Tzu (*n* = 20), Greyhound (*n* = 15), Rafeiro Alentejano (*n* = 15), Portuguese Podengo (*n* = 13), Lhasa Apso (*n* = 11), Siberian Husky (*n* = 7), Thai village dog (*n* = 7), Cocker Spaniel (*n* = 6), Hound Cross (*n* = 6), Pug (*n* = 4), Alaskan Malamute (*n* = 3), Bernese mountain dog (*n* = 3), German Shepherd (*n* = 3), Pomeranian (*n* = 3), Terrier (*n* = 3), Bangkeaw (*n* = 2), Chihuahua (*n* = 2), Dachshund (*n* = 2), Australian cattle dog (*n* = 1), Belgian Tervuren (*n* = 1), Boxer Cross (*n* = 1), French Bulldog (*n* = 1), German Shorthair Pointer (*n* = 1), German Wirehair Pointer (*n* = 1), Irish Wolfhound (*n* = 1), Newfoundland (*n* = 1), and Scottish Deerhound (*n* = 1). The included studies used the following research groups (sample size [n]): (i) healthy dogs [*n* = 166]; (ii) benign and malignant oral carcinomas [*n* = 194]; (iii) dental calculus [*n* = 12]; and (iv) periodontal diseases [*n* = 67]. The mean age ± standard deviation (SD) of dogs ranged from 31.5 ± 33.99 months to 128.83 ± 48.30 months (Table 1).

**Table 1:** Primary features of the included studies

Study; Location	Sample size (n)	Gender distribution	Age of dogs (months)	Sample type; sample amount collected	Sample collection time; collection duration	Total protein concentration measurement
***Healthy dogs-associated proteomics studies***						
(de Sousa-Pereira et al. [52]); Portugal	Healthy = 1	1 M/0F	NR	UWS; NR	NR; 4 min	Bradford assay
(Lucena et al. [46]); Portugal	Healthy = 53	29 M/24F	6–132	UWS; NR SWS; NR	3:30–6:30 p.m.; 2 min	Bradford assay
(Pasha et al. [51]); UK	Healthy = 16	8 M/8F	12–96	UWS; NR	8:00 a.m.; 30 s	BCA
(Sanguansermsri et al. [49]); Thailand	Healthy = 7	NR	12–36	UWS; NR	NR	Bradford assay
(Torres et al. [50]); USA	Healthy = 36	16 M/20F	4–148	UWS; NR	NR; 1 min	BCA
(M. Grant et al. [48]); UK	Healthy = 10	3 M/7F	30–78	UWS & AEP; NR	8:00 a.m.; 30 s	BCA
**Study**	**MS-based approach used**	**Peptide labeling approach**	**Protein verification approach**	**Additional approaches**	**FDR threshold**	**Proteins identified (significant/abundant)**
(de Sousa-Pereira et al. [52])	LC-MS/MS	Label-free	SDS-PAGE	LC-MS/MS	5%	244 (12)
(Lucena et al. [46])	MALDI-TOF-MS	Label-free	SDS-PAGE 2D-PAGE	MALDI-TOF/TOF-MS	NR	16 (5)
(Pasha et al. [51])	LC-MS/MS	TMT	NR	SDS-PAGE	1%	72 (9)
(Sanguansermsri et al. [49])	LC-MS/MS	Label-free	LC-MS/MS	SDS-PAGE	NR	2532 (44)
(Torres et al. [50])	LC-MS/MS	Label-free	Scaffold software	SDS-PAGE	≤ 1%	2491 (10)
(M. Grant et al. [48])	LC-MS/MS	TMT	NR	NR	1%	96 (5)
***Oral diseases-associated proteomics studies***						
**Study; Location**	**Study groups; sample size**	**Gender distribution**	**Age of dogs (months)**	**Sample type; sample amount collected**	**Sample collection time; collection duration**	**Total protein concentration measurement**
(Davis et al. [18]); UK	Mild G; Moderate G; Mild P (Total: 52)	24 M/28F	16–83	GCF; NR	NR; 30 s	NR
(Pisamai et al. [17]); Thailand	EOM = 7; LOM = 8; OSCC = 7; BOT = 8; Healthy = 8	NR	12–192	NR	NR	Lowry assay
(Ploypetch et al. [19]); Thailand	EOM = 5; LOM = 24; OSCC = 10; BOT = 11; *P* = 5; Healthy = 10	NR	84–168	UWS; 500–1000 µL	NR; 5–10 min	Lowry assay
(Bringel et al. [23]); Canada	Healthy = 8; Calculus = 12	7 M/13F	3–108	SWS; 100–1000 µL	NR; 5–10 min	µBCA
(Ploypetch et al. [20]); Thailand	EOM = 5; LOM = 24; OSCC = 10; BOT = 11; *P* = 5; Healthy = 10	36 M/26F	24–180	UWS; 500–1000 µL	NR; 5–10 min	Lowry assay
(Ploypetch et al. [21]); Thailand	OM = 9	4 M/5F	91–157	UWS; NR	NR	Lowry assay
(Ploypetch et al. [22]); Thailand	EOM = 5; LOM = 28; LOSCC = 10; BOT = 12; CP = 5; Healthy = 7	44 M/23F	84–168	Serum; 500 µL	NR	Lowry method
**Study**	**MS-based approach used**	**Peptide labeling approach**	**Protein verification approach**	**Additional approaches**	**FDR threshold**	**Proteins identified (Significant/abundant)**
(Davis et al. [18])	LC-MS/MS	iTRAQ	ELISA	NR	1%	406 (32)
(Pisamai et al. [17])	MALDI-TOF-MS coupled with LC-MS/MS	Label-free	LC-MS/MS	SDS-PAGE	NR	1572 (22)
(Ploypetch et al. [19])	MALDI-TOF-MS coupled with LC-MS/MS	Label-free	Western blot	Western blot	NR	18
(Bringel et al. [23])	LC-MS/MS	Label-free	NR	NR	1%	658 (34)
(Ploypetch et al. [20])	LC-MS/MS	Label-free	Western blot	Western blot	NR	3726 (27)
(Ploypetch et al. [21])	LC-MS/MS	Label-free	Western blot	Western blot	NR	74 (1)
(Ploypetch et al. [22])	MALDI-TOF-MS coupled with LC-MS/MS	Label-free	NR	NR	NR	16 (4)

*Abbreviations*  = µBCA; micro-bicinchoninic acid assay; 2D-PAGE: two-dimensional polyacrylamide gel electrophoresis; BCA: bicinchoninic acid assay; BOT: benign oral tumor; CP: chronic periodontitis; ELISA: enzyme-linked immunosorbent assay; EOM: early-stage oral melanoma; F: female; FDR: false discovery rate; G: gingivitis; GCF: gingival crevicular fluid; iTRAQ: isobaric tags for relative and absolute quantitation; LC-MS/MS: liquid chromatography tandem mass spectrometry; LOM: late-stage oral melanoma; LOSCC: late-stage oral squamous cell carcinoma; M: male; MALDI-TOF-MS: matrix-assisted laser desorption ionization time of flight mass spectrometry; MS: mass spectrometry; NR: no reported; OM: oral melanoma; OSCC: oral squamous cell carcinoma; P: periodontitis; SDS-PAGE: sodium dodecyl sulfate polyacrylamide gel electrophoresis; SWS: stimulated whole saliva; TMT: tandem mass tags; UWS: unstimulated whole saliva

### Sample biofluids collection and total protein concentration measurement

Saliva was the most frequently used sample biofluid with 8 studies using unstimulated whole saliva (UWS; 500–1000 µL) [19–21, 48–52], 1 used stimulated whole saliva (SWS; 100–1000 µL) [23], and 1 used both UWS and SWS [53]. Blood serum (500 µL) [22] and GCF [18] were used as the sample biofluid in one study each, while one study did not report any sample biofluid utilized [17]. Only 3 of 13 studies reported the sample collection time, i.e., 8:00 a.m [48, 51]. and 3:30 p.m. to 6:30 p.m [53]. , while 9 studies reported the duration of sample collection, i.e., 30 s [18, 48, 51], 1 min [50], 2 min [53], 4 min [52], and 5 to 10 min [19, 20, 23]. Varying methods were used to measure the total protein content of the sample biofluid including bicinchoninic acid (BCA) assay [48, 50, 51], micro-BCA (µBCA) [23], Bradford assay [49, 52, 53], and Lowry assay [17, 19–22] (Table 1).

### MS-based proteomics approaches

For proteomics analysis, 9 studies used liquid chromatography tandem MS (LC-MS/MS) [18, 20, 21, 23, 48–52], 1 used matrix-assisted laser desorption ionization time of flight MS (MALDI-TOF-MS) [53], while 3 studies utilized MALDI-TOF-MS coupled with LC-MS/MS [17, 19, 22]. The label-free approach was used by 10 studies, while three studies used tandem mass tag (TMT) [48, 51] and isobaric tags for relative and absolute quantitation (iTRAQ) [18] approaches for peptide labeling. The validation/verification of the corresponding protein(s) was performed in 9 studies using different methods including LC-MS/MS [17, 49], sodium dodecyl sulfate-polyacrylamide gel electrophoresis (SDS-PAGE) [52, 53], two-dimensional (2D)-PAGE [53], enzyme-linked immunosorbent assay (ELISA) [18], western blotting [19–21], and Scaffold software [50]. For protein identification, varying false discovery rate (FDR) threshold values were used including ≤ 1% [50], 1% [18, 23, 48, 51], and 5% [52] (Table 1).

### Proteomics profiling outcomes of healthy dogs

Among the included studies that identified and characterized the proteomics profile of healthy dogs using MS identified a total of 5,451 proteins, of which 137 were reported to be the most abundant (Supplementary File).

#### Gene ontology, KEGG pathway, and protein-protein interaction analysis

**Fig. 2:**
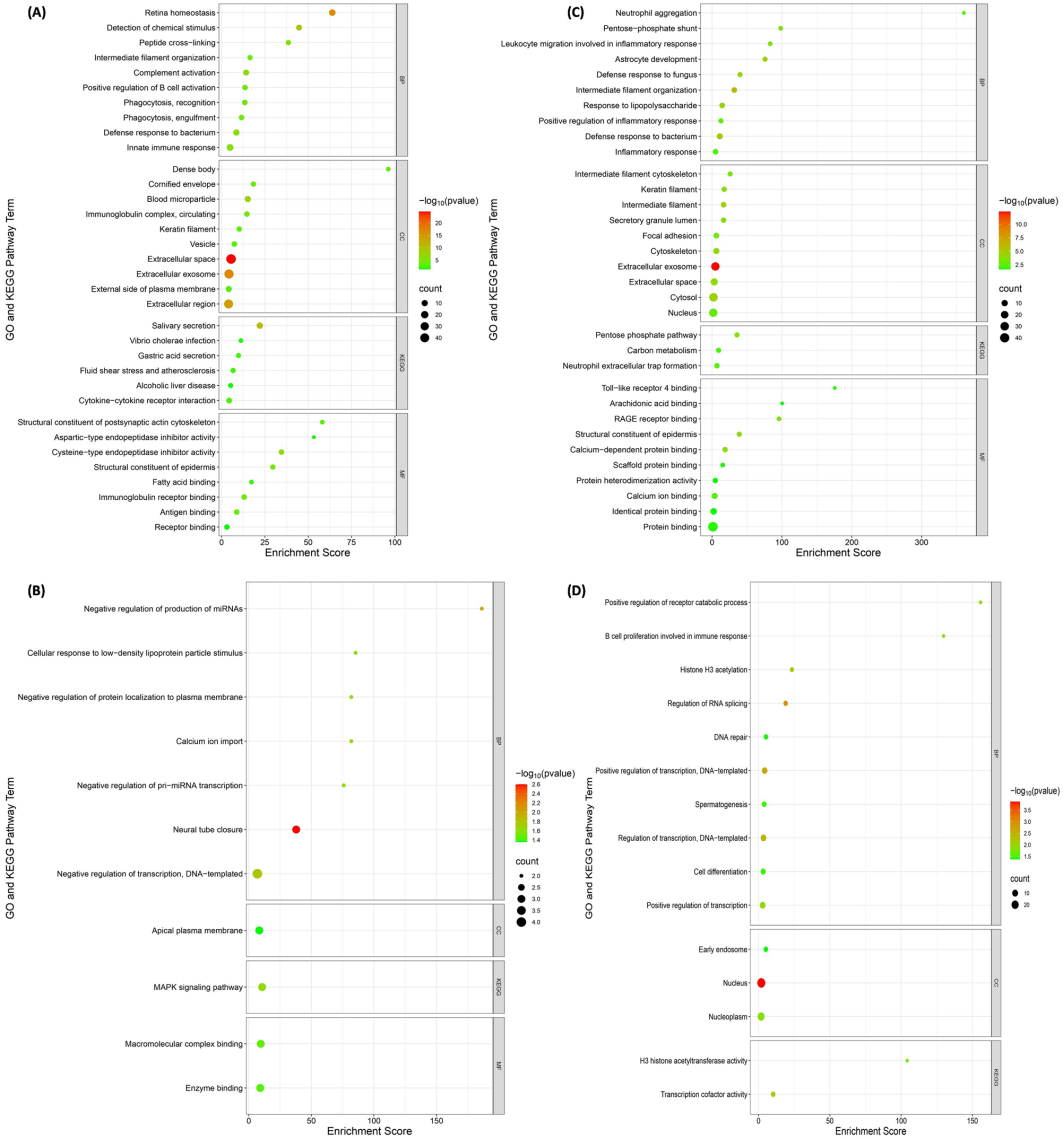
Gene Ontology (GO) enrichment and KEGG pathway analyses of the most abundant proteins found in: (**A**) healthy dogs; (**B**) dogs with dental calculus; (**C**) dogs with periodontal diseases; and (**D**) dogs with oral tumors. These analyses encompassed biological processes (BP), cellular compartments (CC), KEGG pathways, and molecular functions (MF). The X-axis represents GO-KEGG pathway terms, and the Y-axis depicts the enrichment score, reflecting the degree of enrichment based on Fisher’s exact test *p*-values. Node size corresponds to the number of proteins associated, and node color indicates statistical significance

**Fig. 3:**
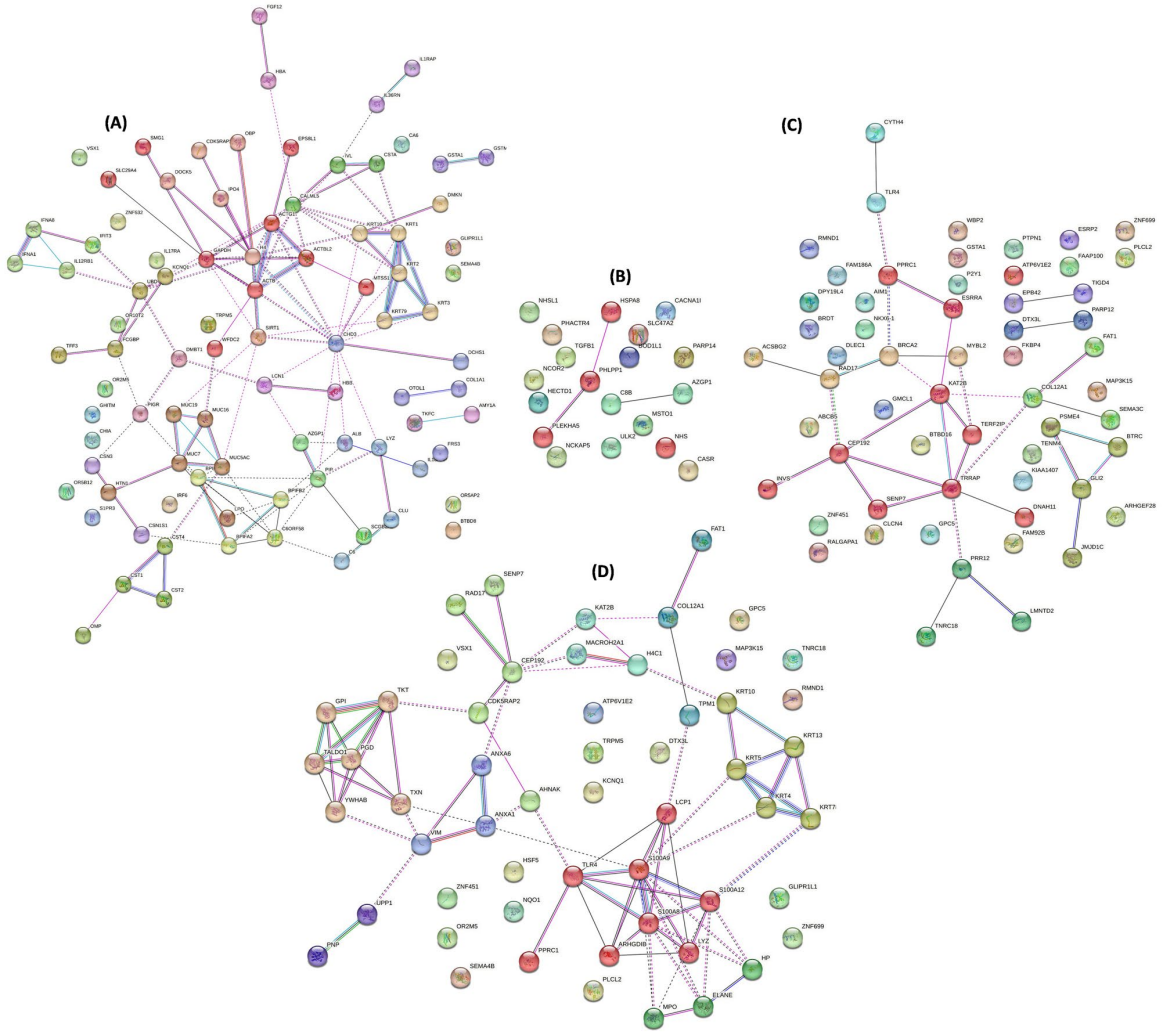
The protein-protein interactions analysis of (**A**) healthy dogs; (**B**) dogs with dental calculus; (**C**) dogs with periodontal diseases; and (**D**) dogs with oral tumors. Nodes displaying dual edges signify their presence in multiple proteins. The diversity in line colors indicates the use of various types of evidence in predicting protein connections

The BP and MF analyses indicated that the majority of the proteins in healthy dogs were involved in ‘innate immune response’ (*p* = 4.10 × 10^− 06^) and ‘cysteine-type endopeptidase inhibitor activity’ (*p* = 7.41 × 10^− 7^), respectively. Most of the proteins were located in the ‘extracellular space’ (53%) and the highest enriched KEGG pathway was ‘salivary secretion’ (*p* = 3.17 × 10^− 11^) (Fig. 2A). Applying MCL clustering resulted in 9 clusters each with at least three genes. The biggest cluster (red), associated with ‘postsynaptic actin cytoskeleton organization’ included the following 9 proteins: ACTB, ACTBL2, ACTG1, EPS8L1, GAPDH, MTSS1, SLC29A4, SMG1, and WFDC2 (Fig. 3A).

### Proteomics profiling outcomes of dogs with oral diseases

A total of 6,470 proteins were identified in dogs with oral diseases, of which 58 were most abundant in dogs with periodontal diseases; 19 in dental calculus; and 63 in oral tumors (Supplementary File).

#### Gene ontology, KEGG pathway, and protein-protein interaction analysis

Regarding the BP analysis, most of the proteins in dogs with periodontal diseases (Fig. 2C), dental calculus (Fig. 2B), and oral tumors (Fig. 2D) were associated with ‘defense response to bacterium’ (*p* = 6.55 × 10^− 06^), ‘negative regulation of transcription, DNA-templated’ (*p* = 0.016), and ‘positive regulation of transcription, DNA-templated’ (*p* = 0.003), respectively. MF analysis demonstrated that the proteins were involved in ‘protein binding’ (*p* = 0.015), ‘macromolecular complex binding’ (*p* = 0.033), and ‘transcription co-factor activity’ (*p* = 0.006), respectively. Most of the proteins were found in ‘cytosol’ (55%), ‘cytoplasm’ (47%), and ‘nucleus’ (52%), respectively. However, the highest enriched KEGG pathways included the ‘neutrophil extracellular trap formation’ (*p* = 0.004) and the ‘MAPK signaling pathway’ (*p* = 0.022) in dogs with periodontal diseases and dental calculus, respectively. For dogs with periodontal diseases (Fig. 3C), dental calculus (Fig. 3B), and oral tumors (Fig. 3D), the application of MCL clustering formed 8, 1, and 5 clusters, respectively, each with at least three genes. The biggest clusters in dogs with oral diseases were: (i) periodontal diseases: ARHGDIB, LCP1, LYZ, PPRC1, S100A8, S100A9, S100A12, and TLR4 [associated with ‘defense response to bacterium’]; (ii) dental calculus: HSPA8, PHLPP1, and PLEKHA5; and (iii) oral tumors: CEP192, DNAH11, ESRRA, INVS, KAT2B, PPRC1, SENP7, TERF2IP, and TRRAP [involved in ‘positive regulation of transcription, DNA-templated].

### Risk of bias outcomes

**Fig. 4:**
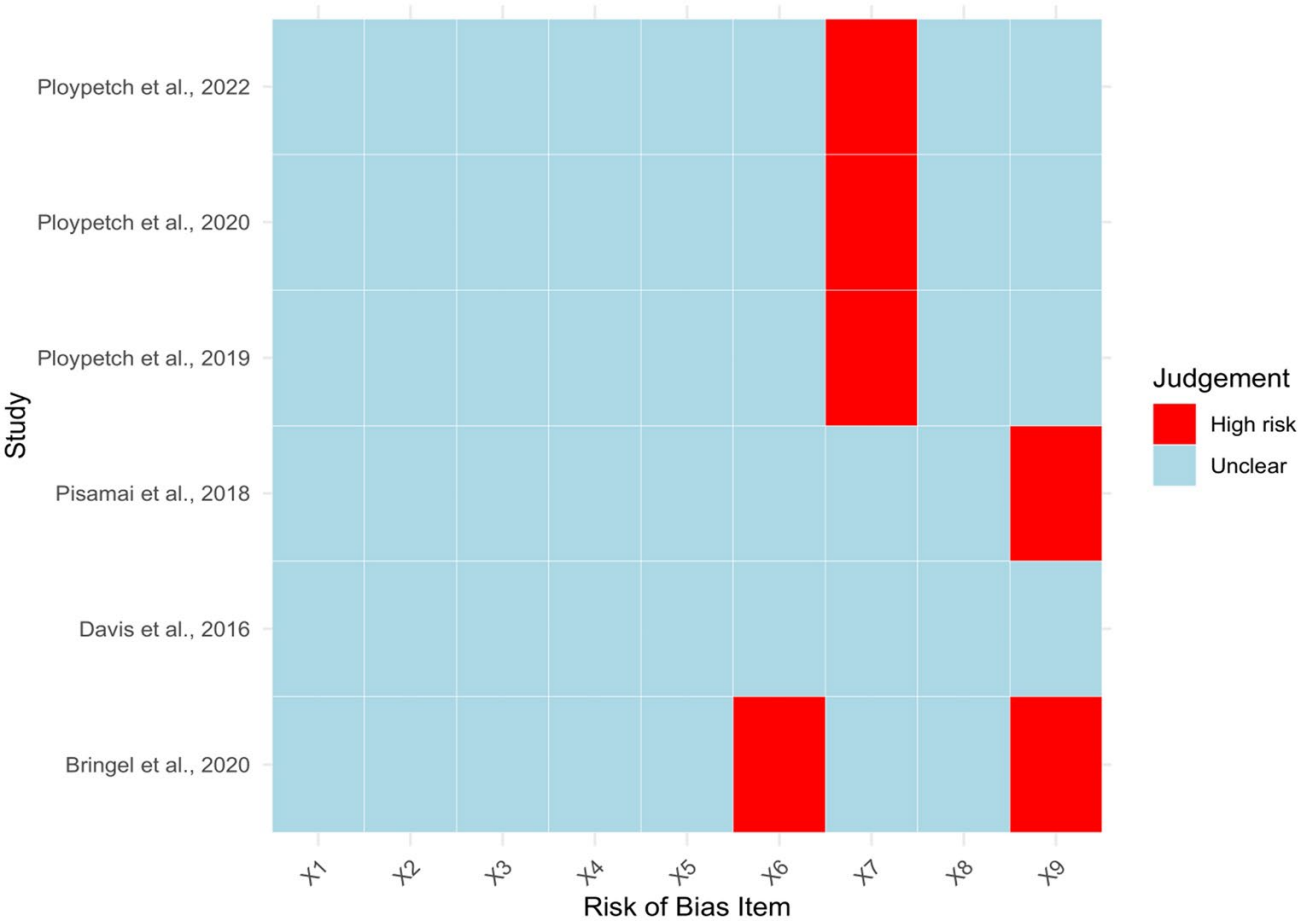
The summary of the risk of bias assessed in the included studies. In this context, red color denotes a high risk, while light blue color signifies a low risk. X1 = was the sample representative of the target population; X2 = were study participants recruited in an appropriate manner; X3 = was the sample size adequate; X4 = were the research participants and the setting described in detail; X5 = was the data analysis performed with adequate coverage of the identified sample; X6 = were objective and standard criteria utilized to measure the condition; X7 = was the disease measured reliably; X8 = was there appropriate statistical/data analysis; and X9 = are all important confounding factors, subgroups, differences identified and accounted for. The figure was made using the *ggplot2* and *tidyr* functions of RStudio.

Only six studies were provided to the JBI Critical Appraisal Checklist for Studies Reporting Prevalence Data, as they encompassed case-control evaluations that compared healthy dogs to dogs with oral disease(s) [17–20, 22, 23]. All included studies were found to have low RoB (high quality) (Fig. 4).

## Discussion

This systematic review aimed to investigate the similarities and differences between the oral biofluids’ proteomics profile of dogs with and without oral diseases. The study revealed significant differences between the dogs with and without oral diseases including the number of proteins identified, most abundant proteins, cellular localization, molecular and biological functions, KEGG pathways, and PPIs.

Humans and companion animals often experience similar diseases, including periodontal diseases, obesity, renal disorders, cardiovascular diseases, and cancer [54] rendering them valuable subjects for comparative proteomics investigations. Dogs exhibit comparable anatomical and physiological features to humans and coexist in the same living environment as their owners. Their relatively short lifespans, coupled with the provision of advanced veterinary care, and the restricted genetic diversity, particularly in purebred dogs susceptible to inherited conditions mirroring various human genetic disorders, position them as noteworthy models for certain genetic and oral diseases [25, 37].

In this systematic review, we have included studies that encompass a variety of dog breeds. The influence of different dog breeds on proteomics analyses of dogs both with and without oral diseases can have a significant and complex impact. To begin with, the genetic variations present among different dog breeds can influence the patterns of protein expression, potentially resulting in disparities in the observed proteomic profiles between breeds [23]. This variation may have implications for the identification of biomarkers specific to certain diseases and the interpretation of data derived from proteomics. Furthermore, distinctions in susceptibility to oral diseases, such as periodontal disease or oral tumors, based on breed can affect the composition and abundance of proteins found in oral tissues or fluids. Breed-specific anatomical characteristics, composition of oral microbiota, and environmental factors can also contribute to the variability in proteomic signatures associated with oral health and disease [51]. Additionally, the availability of breed-specific reference databases for proteomics analysis may differ, impacting the accuracy and comprehensiveness of protein identification and quantification. It is crucial to comprehend and take into account these breed-related factors in order to ensure the robustness and generalizability of proteomics studies in dogs with oral diseases. This understanding will facilitate the development of personalized diagnostic and therapeutic approaches tailored to the specific breeds of canines.

Saliva, GCF, and serum were employed as sample biofluid among the included studies. The selection of biofluids for proteomics investigations in canines introduces various factors to be taken into account and trade-offs to be made. Analysis of serum provides valuable insights into systemic alterations, reflecting overall physiological conditions such as inflammation and metabolism. However, due to its systemic nature, it may have limitations in directly capturing local changes specific to oral tissues [55]. In contrast, GCF offers a more localized perspective, containing proteins from the gingival tissue and immune cells within the oral environment. Despite its relevance, the collection of GCF can pose technical challenges, and its composition may vary depending on factors such as the technique of sampling and the severity of the disease [56]. Saliva analysis emerges as a non-invasive and easily accessible option, reflecting both systemic and local changes in the oral cavity [57]. However, careful consideration is necessary due to its susceptibility to variability caused by factors such as salivary flow rate and oral hygiene practices [58]. Each biofluid has distinct advantages and challenges, underscoring the significance of aligning the selection of biofluid with the research objectives and methodological considerations in order to maximize the insights gained from proteomics analyses in oral diseases of canines.

The choice of saliva type, whether it is stimulated or unstimulated, when evaluating biomarkers in proteomics studies of dogs with and without oral diseases, carries significant implications for the outcomes and interpretations of research. Stimulated saliva, which is produced in response to mechanical or gustatory stimuli, offers certain advantages such as an increased rate and volume of flow, potentially enhancing the sensitivity of proteomic analyses [59]. However, the collection of stimulated saliva may introduce variability due to the requirement for external stimuli, and the higher flow rate could dilute specific biomarkers [60]. On the other hand, unstimulated saliva represents the baseline saliva production state, providing a stable foundation for the analysis of biomarkers without any external influences. While it is relatively easy to collect and exhibits stability, unstimulated saliva may have lower concentrations of biomarkers and a limited range of variability compared to its stimulated counterpart [61]. Understanding the trade-offs between these two types of saliva is essential for researchers, as the choice made directly impacts the sensitivity, reliability, and clinical significance of proteomic findings in oral diseases of dogs. By incorporating both types of saliva and utilizing complementary analytical strategies, the comprehensiveness and dependability of proteomics studies can be enhanced, leading to a deeper understanding of oral health in dogs and facilitating the development of diagnostic and therapeutic approaches.

The findings of this review found that most of the proteins present in healthy dogs were associated with ‘innate immune response’. The importance of proteins linked to the biological function of ‘innate immune response’ resides in their vital function as the initial defense against invading pathogens and their role in governing intricate signaling and transcriptional networks [62]. The innate immune response encompasses a variety of interactions, including protein-protein and DNA-protein interactions, as well as signaling cascades, underscoring its multifaceted nature. This response extends beyond straightforward linear pathways, embracing intricate networks of molecular interactions and transcriptional responses [63].

The most abundant proteins in dogs with periodontal diseases were involved in the ‘defense response to bacterium’. The significance of proteins associated with ‘defense response to bacterium’ lies in their pivotal role in initiating inflammatory host responses and shaping the immune reaction to bacterial infection. These processes are central to the development of periodontal diseases [64]. The inflammatory host responses triggered by bacteria, coupled with the direct deleterious effects of the bacteria, contribute predominantly to the tissue damage observed in periodontal diseases [65]. The interplay between periodontal inflammation and persistent bacterial infection elevates the expression and activity of neutral proteinases, further contributing to the observed tissue destruction [66]. Moreover, the imbalances in the immune response, coupled with uncontrolled inflammation, have been implicated in the tissue damage seen in periodontitis, underscoring the critical involvement of the host immune response in the pathogenesis of periodontal diseases [67].

The majority of the proteins in dogs with dental calculus were involved in the ‘negative regulation of transcription’. Proteins associated with the negative regulation of transcription may contribute to the modulation of gene expression linked to inflammatory mediators and immune responses. Dental calculus is linked to persistent inflammation in the surrounding tissues, and the disruption of the immune response is implicated in the development of periodontal diseases, closely associated with dental calculus [68]. Consequently, proteins involved in transcriptional regulation may exert an influence on the expression of genes associated with the immune response and inflammation, potentially influencing the advancement and severity of conditions related to dental calculus [23].

In dogs with oral tumors, most of the proteins were associated with ‘positive regulation of transcription’ lies in their pivotal role in enhancing the transcription of genes. This action can impact diverse cellular processes and contribute to the initiation and progression of tumorigenesis [69]. The dysregulation of transcriptional processes is a distinctive feature of cancer, and positive regulation of transcription can result in the heightened expression of genes crucial for cell proliferation, survival, and metastasis – key elements in the development and advancement of tumors [70]. Transcriptional dysregulation, including positive regulation, is intimately associated with the onset and progression of various cancers, including oral tumors [71].

Animal proteomics finds application in leveraging domestic animal research as a model to unravel pathways in humans. Additionally, utilizing animal proteomics to address human research inquiries holds promise, facilitating the horizontal transfer of knowledge. The effective exploitation of such data in bioinformatics requires adept use of tools, databases, and the corresponding skills. A notable gap exists in the availability of trained bioinformaticians in the field of animal sciences, necessitating investment in training, knowledge enhancement, and experience sharing. To propel progress efficiently, the recommendation is to establish specialized services encompassing individuals, software, and hardware dedicated to bioinformatics. This approach ensures the streamlined management of horizontal activities across diverse research projects in animal proteomics. The ongoing advancement of high-throughput proteomics in animal sciences is poised to benefit significantly from the application of bioinformatics. This synergy promises to have a positive and substantial impact not only on animal sciences but also on human research.

An ongoing challenge in research, particularly with the swift progress in quantitative proteomics, is the imperative for result validation before complete acceptance. Various methods are employed to substantiate the measurement of proteins, whether through relative or absolute proteomics. The primary approach involves the use of antibody-based techniques including Western blot or ELISA. In animal proteomics, a limitation arises in these validation procedures due to the absence of species-specific antibodies. Nevertheless, whenever a suitable antibody is accessible, it becomes crucial to validate results, thereby affirming their accuracy and reliability.

Conducting a meta-analysis of proteomics investigations in canines affected by oral diseases poses noteworthy difficulties. The wide array of study designs and methodologies introduces heterogeneity, thereby complicating comparisons and synthesis. Furthermore, the scarcity of data pertaining specifically to oral diseases in canines limits the thoroughness of the examination. Ensuring the quality and standardization of data is imperative; however, inconsistent reporting standards impede interpretation. Additionally, the variability in disease phenotypes and sample types adds complexity. Addressing bias and confounding factors is crucial, given the disparities in sample populations and environmental influences. In this systematic review, one of the primary limitations we encountered pertained to the feasibility of conducting a meta-analysis. The objective was to employ a meta-analysis to thoroughly analyze the proteomics investigations. Nevertheless, we faced a significant obstacle inherent to the nature of proteomics meta-analysis. Unlike traditional meta-analyses, which frequently utilize effect size measures such as means or odds ratios, conducting a meta-analysis for proteomics investigations necessitates access to fold change values of proteins that are differentially expressed across multiple inquiries. Unfortunately, despite exhaustive efforts, we encountered a challenge in obtaining the necessary data for our analysis. Specifically, we were unable to identify two or more differentially expressed proteins reported across two or more investigations within our included studies. Consequently, the lack of sufficient data prevented us from carrying out the intended meta-analysis.

Given the shared susceptibility of humans and companion animals to similar diseases [57], dogs serve as valuable models for comparative proteomics investigations, owing to their analogous anatomical and physiological features, cohabitation with humans, and genetic predispositions to certain conditions mirroring human disorders. The study findings shed light on the pivotal role of proteins associated with innate immune responses in healthy dogs, defense responses to bacteria in periodontal diseases, negative regulation of transcription in dental calculus, and positive regulation of transcription in oral tumors. Leveraging animal proteomics, particularly in domestic animals such as dogs, holds promise for advancing human and veterinary research through knowledge transfer and bioinformatics applications [57]. However, challenges persist in result validation, data interpretation, and meta-analysis feasibility, underscoring the need for continued efforts to enhance research methodologies and interdisciplinary collaboration in animal proteomics studies.

## Conclusion

This systematic review unveiled significant differences in the proteomics profiles of oral biofluids between dogs with and without oral diseases. The synergy of animal proteomics and bioinformatics offers a promising avenue for cross-species research, despite persistent challenges in result validation. The continuous advancement of high-throughput proteomics in animal sciences, guided by bioinformatics, holds potential for substantial impacts on both animal and human research.

### Electronic supplementary material

Below is the link to the electronic supplementary material.

Supplementary Material 1: Supplementary Table 1: List of the most abundant proteins reported in the included studies. Green highlight represents the most abundant proteins found in healthy dogs; Orange highlight represents the most abundant proteins found in healthy dogs and dogs with periodontal diseases; Yellow highlight represents the most abundant proteins found in dogs with benign oral tumors; Light blue highlight represents the most abundant proteins found in dogs with early-stage oral melanoma; Purple highlight represents the most abundant proteins found in dogs with late-stage oral melanoma; Red highlight represents the most abundant proteins found in dogs with oral squamous cell carcinoma; Grey highlight represents the most abundant proteins found in dogs with periodontal diseases, benign oral tumors, early-stage oral melanoma; late-stage oral melanoma, and oral squamous cell carcinoma; Dark red highlight represents the most abundant proteins found in dogs with dental calculus; Light blue highlight represents the most abundant proteins found in dogs with mixed cancers; and Orange red highlight represents the most abundant proteins found in dogs with periodontal diseases.Supplementary Table 2: List of names of all breeds of dogs used in the included studies along with the number of dogs used. Supplementary Table 3: Gene Ontology analysis – biological processes of the most abundant proteins among healthy dogs identified in the included studies. Supplementary Table 4: Gene Ontology analysis – cellular compartments of the most abundant proteins among healthy dogs identified in the included studies.Supplementary Table 5: Gene Ontology analysis – molecular functions of the most abundant proteins among healthy dogs identified in the included studies. Supplementary Table 6: KEGG pathway analysis of the most abundant proteins among healthy dogs identified in the included studies. Supplementary Table 7: Gene Ontology analysis – biological processes of the most abundant proteins among periodontitis dogs identified in the included studies. Supplementary Table 8: Gene Ontology analysis – cellular compartments of the most abundant proteins among periodontitis dogs identified in the included studies. Supplementary Table 9: Gene Ontology analysis – molecular functions of the most abundant proteins among periodontitis dogs identified in the included studies. Supplementary Table 10: KEGG pathway analysis of the most abundant proteins among periodontitis dogs identified in the included studies. Supplementary Table 11: Gene Ontology analysis – biological processes of the most abundant proteins among dogs with calculus identified in the included studies. Supplementary Table 12: Gene Ontology analysis – cellular compartments of the most abundant proteins among dogs with calculus identified in the included studies.Supplementary Table 13: Gene Ontology analysis – molecular functions of the most abundant proteins among dogs with calculus identified in the included studies.Supplementary Table 14: KEGG pathway analysis of the most abundant proteins among dogs with calculus identified in the included studies.Supplementary Table 15: Gene Ontology analysis – biological processes of the most abundant proteins among dogs with oral tumors identified in the included studies.Supplementary Table 16: Gene Ontology analysis – cellular compartments of the most abundant proteins among dogs with oral tumors identified in the included studies.Supplementary Table 17: Gene Ontology analysis – molecular functions of the most abundant proteins among dogs with oral tumors identified in the included studies.Supplementary Table 18: Protein-Protein interactions analysis of the most abundant proteins among healthy dogs identified in the included studies. Supplementary Table 19: Protein-Protein interactions analysis of the most abundant proteins among dogs with calculus identified in the included studies. Supplementary Table 20: Protein-Protein interactions analysis of the most abundant proteins among periodontitis dogs identified in the included studies. Supplementary Table 21: Protein-Protein interactions analysis of the most abundant proteins among dogs with oral tumors identified in the included studies.

## Data Availability

Data is provided within the manuscript or supplementary information files.

## Abbreviations

µBCA: micro-BCA
ACTB: Actin, cytoplasmic 1
ACTBL2: Beta-actin-like protein 2
ACTG1: Actin, cytoplasmic 2
ARHGDIB: Rho GDP-dissociation inhibitor 2
BCA: bicinchoninic acid
BP: biological processes
CC: cellular compartments
CEP192: Centrosomal protein of 192 kDa
DAVID: Database for Annotation, Visualization, and Integrated Discovery
DNAH11: Dynein axonemal heavy chain 11
EPS8L1: Epidermal growth factor receptor kinase substrate 8-like protein 1
ESRRA: Steroid hormone receptor ERR1
GAPDH: Glyceraldehyde-3-phosphate dehydrogenase
GCF: gingival crevicular fluid
HSPA8: Heat shock cognate 71 kDa protein
INVS: Inversin
JBI: Joanna Briggs Institute
KAT2B: Histone acetyltransferase KAT2B
KEGG: Kyoto Encyclopedia of Genes and Genomes
LCP1: Plastin-2
LYZ: Lysozyme C
MCL: Markov Cluster algorithm
MeSH: Medical Subject Headings
MF: molecular functions
MTSS1: Protein MTSS 1
OSF: Open Science Framework
PHLPP1: PH domain leucine-rich repeat-containing protein phosphatase 1
PICO: Population, Intervention, Comparison, Outcome
PLEKHA5: Pleckstrin homology domain-containing family A member 5
PPI: protein-protein interaction
PPRC1: Peroxisome proliferator-activated receptor gamma coactivator-related protein 1
PRISMA: Preferred Reporting Items for Systematic Reviews and Meta-Analyses
S100A12: Protein S100-A12
S100A8: Protein S100-A8
S100A9: Protein S100-A9
RoB: Risk of bias
SENP7: Sentrin-specific protease 7
SLC29A4: Equilibrative nucleoside transporter 4
SMG1: Serine/threonine-protein kinase SMG1
STRING: Search Tool for the Retrieval of Interacting Genes/Proteins
SWS: stimulated whole saliva
TERF2IP: Telomeric repeat-binding factor 2-interacting protein 1
TLR4: Toll-like receptor 4
TMT: tandem mass tag
TRRAP: Transformation/transcription domain-associated protein
UWS: unstimulated whole saliva
WFDC2: WAP four-disulfide core domain protein 2

## References

[1] Hughes J, Macdonald DW (2013). A review of the interactions between free-roaming domestic dogs and wildlife. Biol Conserv.

[2] O’haire ME, Rodriguez KE (2018). Preliminary efficacy of service dogs as a complementary treatment for posttraumatic stress disorder in military members and veterans. J Consult Clin Psychol.

[3] Worth A, Cave N (2018). A veterinary perspective on preventing injuries and other problems that shorten the life of working dogs. Rev Sci Tech Int off Epizoot.

[4] Chapagain D, Range F, Huber L, Virányi Z (2018). Cognitive aging in dogs. Gerontology.

[5] Switonski M (2014). Dog as a model in studies on human hereditary diseases and their gene therapy. Reprod Biol.

[6] Powers JC, Recchia F. Canine model of pacing-induced heart failure. Methods Mol Biol 2018:309–25. 10.1007/978-1-4939-8597-5_24 29987830

[7] Hayward JJ, Castelhano MG, Oliveira KC (2016). Complex disease and phenotype mapping in the domestic dog. Nat Commun.

[8] Schlieben P, Meyer A, Weise C (2012). Differences in the proteome of high-grade versus low-grade canine cutaneous mast cell tumours. Vet J.

[9] Thanomsridetchai N, Singhto N, Tepsumethanon V (2011). Comprehensive proteome analysis of hippocampus, brainstem, and spinal cord from paralytic and furious dogs naturally infected with rabies. J Proteome Res.

[10] Burgess K, Burchmore R (2012). Strategies to dissect parasite proteomes. Parasitology.

[11] Corthals GL, Wasinger VC, Hochstrasser DF, Sanchez JC (2000). The dynamic range of protein expression: a challenge for proteomic research. Electrophoresis.

[12] Ceciliani F, Eckersall D, Burchmore R, Lecchi C (2014). Proteomics in veterinary medicine: applications and trends in disease pathogenesis and diagnostics. Vet Pathol.

[13] Soares R, Franco C, Pires E (2012). Mass spectrometry and animal science: protein identification strategies and particularities of farm animal species. J Proteom.

[14] Bai Y, Sartor M, Cavalcoli J (2012). Current status and future perspectives for sequencing livestock genomes. J Anim Sci Biotechnol.

[15] Tholey A, Taylor NL, Heazlewood JL, Bendixen E (2017). We are not alone: the iMOP initiative and its roles in a biology-and disease-driven human proteome project. J Proteome Res.

[16] Bilić P, Kuleš J, Galan A (2018). Proteomics in veterinary medicine and animal science: neglected scientific opportunities with immediate impact. Proteomics.

[17] Pisamai S, Roytrakul S, Phaonakrop N, Jaresitthikunchai J, Suriyaphol G (2018). Proteomic analysis of canine oral tumor tissues using MALDI-TOF mass spectrometry and in-gel digestion coupled with mass spectrometry (GeLC MS/MS) approaches. PLoS ONE.

[18] Davis IJ, Jones AW, Creese AJ, Staunton R, Atwal J, Chapple IL (2016). Longitudinal quantification of the gingival crevicular fluid proteome during progression from gingivitis to periodontitis in a canine model. J Clin Periodontol.

[19] Ploypetch S, Roytrakul S, Jaresitthikunchai J, Phaonakrop N, Krobthong S, Suriyaphol G (2019). Salivary proteomics of canine oral tumors using MALDI-TOF mass spectrometry and LC-tandem mass spectrometry. PLoS ONE.

[20] Ploypetch S, Roytrakul S, Phaonakrop N, Kittisenachai S, Leetanasaksakul K, Pisamai S (2020). In-gel digestion coupled with mass spectrometry (GeLC-MS/MS)-based salivary proteomic profiling of canine oral tumors. BMC Vet Res.

[21] Ploypetch S, Roytrakul S, Jaresitthikunchai J, Phaonakrop N, Teewasutrakul P, Rungsipipat A (2021). Salivary proteomics in monitoring the therapeutic response of canine oral melanoma. PLoS ONE.

[22] Ploypetch S, Jaresitthikunchai J, Phaonakrop N, Sakcamduang W, Manee-In S, Suriyaphol P (2022). Utilizing MALDI-TOF MS and LC-MS/MS to access serum peptidome-based biomarkers in canine oral tumors. Sci Rep.

[23] Bringel M, Jorge PK, Francisco PA (2020). Salivary proteomic profile of dogs with and without dental calculus. BMC Vet Res.

[24] Bendixen E, Danielsen M, Hollung K, Gianazza E, Miller I (2011). Farm animal proteomics—a review. J Proteom.

[25] Doherty MK, Beynon RJ, Whitfield PD (2008). Proteomics and naturally occurring animal diseases: opportunities for animal and human medicine. Proteom Clin Appl.

[26] Kaneko JJ, Harvey JW, Bruss ML. Clinical biochemistry of domestic animals. Academic; 2008.

[27] Eckersall PD, McLaughlin M. Proteomics in animal health and disease. Methods Anim Proteom 2011:243–318.

[28] Henry CJ (2010). Biomarkers in veterinary cancer screening: applications, limitations and expectations. Vet J.

[29] Kirby G, Mackay A, Grant A (2011). Concentration of lipocalin region of collagen XXVII alpha 1 in the serum of dogs with hemangiosarcoma. J Vet Intern Med.

[30] Locatelli C, Piras C, Riscazzi G (2016). Serum proteomic profiles in CKCS with mitral valve disease. BMC Vet Res.

[31] Martinez-Subiela S, Horvatic A, Escribano D (2017). Identification of novel biomarkers for treatment monitoring in canine leishmaniosis by high-resolution quantitative proteomic analysis. Vet Immunol Immunopathol.

[32] Escribano D, Cihan H, Martinez-Subiela S (2017). Changes in serum proteins in dogs with Ehrlichia canis infection. Microb Pathog.

[33] Franco-Martínez L, Tvarijonaviciute A, Horvatić A (2018). Changes in salivary analytes in canine parvovirus: a high-resolution quantitative proteomic study. Comp Immunol Microbiol Infect Dis.

[34] Franco-Martínez L, Horvatić A, Gelemanović A (2020). Changes in the salivary proteome associated with canine pyometra. Front Vet Sci.

[35] Miller I, Preßlmayer-Hartler A, Wait R (2014). In between—proteomics of dog biological fluids. J Proteom.

[36] Miller I, Schlosser S, Palazzolo L, Veronesi MC, Eberini I, Gianazza E (2020). Some more about dogs: proteomics of neglected biological fluids. J Proteom.

[37] González-Arostegui LG, Rubio CP, Cerón JJ, Tvarijonaviciute A, Muñoz-Prieto A (2022). Proteomics in dogs: a systematic review. Res Vet Sci.

[38] Ceciliani F, Roccabianca P, Giudice C, Lecchi C (2016). Application of post-genomic techniques in dog cancer research. Mol BioSyst.

[39] Page MJ, McKenzie JE, Bossuyt PM (2021). The PRISMA 2020 statement: an updated guideline for reporting systematic reviews. BMJ.

[40] Methley AM, Campbell S, Chew-Graham C, McNally R, Cheraghi-Sohi S (2014). PICO, PICOS and SPIDER: a comparison study of specificity and sensitivity in three search tools for qualitative systematic reviews. BMC Health Serv Res.

[41] Huang H, Sherman DW, Lempicki BT (2009). Bioinformatics enrichment tools: paths toward the comprehensive functional analysis of large gene lists. Nucleic Acids Res.

[42] Franceschini A, Szklarczyk D, Frankild S (2012). STRING v9. 1: protein-protein interaction networks, with increased coverage and integration. Nucleic Acids Res.

[43] Enright AJ, Van Dongen S, Ouzounis CA (2002). An efficient algorithm for large-scale detection of protein families. Nucleic Acids Res.

[44] Munn Z, Moola S, Riitano D, Lisy K (2014). The development of a critical appraisal tool for use in systematic reviews addressing questions of prevalence. Int J Health Policy Manag.

[45] Franco-Martínez L, Gelemanović A, Horvatić A (2020). The serum and saliva proteome of dogs with diabetes mellitus. Animals.

[46] Lucena S, Carreira MC, Rodrigues L, Capela e Silva F, Tvarijonaviciute A, Lamy E (2018). Comparison of protein precipitation methods for two-dimensional electrophoresis of dog salivary proteins. J Integr Omics.

[47] Polovic N, Waden K, Binnmyr J (2013). Dog saliva–an important source of dog allergens. Allergy.

[48] Grant M, Pasha S, Inui T, Chapple I, Harris S, Holcombe L (2022). A mass spectrometric approach to the proteomic profiling of the Canis lupus familiaris acquired enamel pellicle on hydroxyapatite discs. J Vet Dent.

[49] Sanguansermsri P, Jenkinson HF, Thanasak J (2018). Comparative proteomic study of dog and human saliva. PLoS ONE.

[50] Torres SM, Furrow E, Souza CP (2018). Salivary proteomics of healthy dogs: an in depth catalog. PLoS ONE.

[51] Pasha S, Inui T, Chapple I, Harris S, Holcombe L, Grant MM (2018). The saliva proteome of dogs: variations within and between breeds and between species. Proteomics.

[52] de Sousa-Pereira P, Cova M, Abrantes J (2015). Cross‐species comparison of mammalian saliva using an LC–MALDI based proteomic approach. Proteomics.

[53] Lucena S, Coelho AV, Capela-Silva F, Tvarijonaviciute A, Lamy E (2018). The effect of breed, gender, and acid stimulation in dog saliva proteome. BioMed Res Int.

[54] Freeman LM, Rush JE, Stern JA, Huggins GS, Maron MS (2017). Feline hypertrophic cardiomyopathy: a spontaneous large animal model of human HCM. Cardiol Res.

[55] Fernández-Olavarría A, Mosquera-Pérez R, Díaz-Sánchez R-M, Serrera-Figallo M-A, Gutiérrez-Pérez J-L, Torres-Lagares D (2016). The role of serum biomarkers in the diagnosis and prognosis of oral cancer: a systematic review. J Clin Exp Dent.

[56] Bibi T, Khurshid Z, Rehman A, Imran E, Srivastava KC, Shrivastava D (2021). Gingival crevicular fluid (GCF): a diagnostic tool for the detection of periodontal health and diseases. Molecules.

[57] Ahmad P, Marin LM, Lowe C, Katselis GS, Siqueira WL. Salivary protein homology between humans and dogs: Mass Spectrometry-based proteomics Analysis. J Dent 2024:104855. 10.1016/j.jdent.2024.104855 38246308

[58] Ahmad P, Siqueira WL. Polymorphism of salivary proteins and risk of periodontal diseases: a systematic review and meta-analysis of clinical studies. J Dent 2023:104804. 10.1016/j.jdent.2023.104804 38122885

[59] Ahmad P, Hussain A, Siqueira WL. Mass spectrometry-based proteomic approaches for salivary protein biomarkers discovery and dental caries diagnosis: a critical review. Mass Spectrom Rev 2022:e21822. 10.1002/mas.21822 36444686

[60] Ahmad P, Hussain A, Carrasco-Labra A, Siqueira WL (2022). Salivary proteins as dental caries biomarkers: a systematic review. Caries Res.

[61] Moussa DG, Ahmad P, Mansour TA, Siqueira WL (2022). Current state and challenges of the global outcomes of dental caries research in the meta-omics era. Front Cell Infect Microbiol.

[62] Lynn DJ, Chan C, Naseer M, Yau M, Lo R, Sribnaia A (2010). Curating the innate immunity interactome. BMC Syst Biol.

[63] Elzawahry A, Patil A, Kumagai Y, Suzuki Y, Nakai K (2014). Innate immunity interactome dynamics. Gene Regul Syst Biol.

[64] Genco RJ (1992). Host responses in periodontal diseases: current concepts. J Periodontol.

[65] Pan W, Wang Q, Chen Q (2019). The cytokine network involved in the host immune response to periodontitis. Int J Oral Sci.

[66] Bezerra B, Monajemzadeh S, Silva D, Pirih FQ (2022). Modulating the Immune response in Periodontitis. Front Dent Med.

[67] Kirkwood KL, Cirelli JA, Rogers JE, Giannobile WV (2007). Novel host response therapeutic approaches to treat periodontal diseases. Periodontol 2000.

[68] Silva N, Abusleme L, Bravo D (2015). Host response mechanisms in periodontal diseases. J Appl Oral Sci.

[69] Boyson SP, Gao C, Quinn K (2021). Functional roles of bromodomain proteins in cancer. Cancers.

[70] Anshabo AT, Milne R, Wang S, Albrecht H (2021). CDK9: a comprehensive review of its biology, and its role as a potential target for anti-cancer agents. Front Oncol.

[71] Todd R, Hinds P, Munger K (2002). Cell cycle dysregulation in oral cancer. Crit Rev Oral Biol Med.

